# Prevalence of Middle Ear Infections and Associated Risk Factors in Children under 5 Years in Gasabo District of Kigali City, Rwanda

**DOI:** 10.1155/2017/4280583

**Published:** 2017-12-03

**Authors:** Kaitesi Batamuliza Mukara, Richard J. Lilford, Debara Lyn Tucci, Peter Waiswa

**Affiliations:** ^1^ENT Department, College of Medicine and Health Sciences, University of Rwanda, Kigali, Rwanda; ^2^Department of Health Policy, Planning and Management, Makerere University School of Public Health, Kampala, Uganda; ^3^Warwick Medical School, University of Warwick, Coventry, UK; ^4^Head and Neck Surgery & Communication Sciences, Duke University, Durham, NC, USA; ^5^Global Health Division, Karolinska Institutet, Stockholm, Sweden

## Abstract

Middle ear infections are common in children, and delay in diagnosis and treatment may result in complications such as delays in speech and language development and deafness. The aim of this study was to determine the prevalence and care seeking behaviour for middle ear infections in children under five years in Kigali city. We conducted a cross-sectional study among 810 children aged 6–59 months in Gasabo district of Kigali city, Rwanda. The prevalence of middle ear infections was 5.8%, of whom 4% had chronic suppurative otitis media. A child was less likely to develop middle ear infections if they lived in an urban setting (OR = 0.52, 95% CI: 0.285–0.958) but more likely to develop middle ear infections if exposed to household smoke (OR = 2.54, 95% CI: 1.18–5.46). Parents were unlikely to know that their child had an ear infection (OR: 0.15, 95% CI: 0.06–0.34). Middle ear infection remains a public health problem in Rwanda but many parents were not aware of its presence in the affected children. There is a need to raise awareness of parents about ear infection and to promote early care seeking from qualified health workers.

## 1. Introduction

Infections of the middle ear cleft are common in children. More than half of children will have suffered at least one attack by their third birthday [1–3]. Chronic suppurative otitis media (CSOM) is a middle ear disease entity whose definition is controversial. While the World Health Organization defines CSOM as ear discharge lasting 2 weeks or more through a persistent tympanic membrane perforation [4], otolaryngologists define the duration of discharge as more than 6 weeks [5, 6]. This condition is estimated to affect between 65 and 330 million people worldwide, 60% of whom have significant hearing loss [4]. The majority of those affected reside in low income countries [6]. This prevalence is far higher than the threshold used by the World Health Organization to qualify as a public health problem [7]. There is a varying prevalence of CSOM among African countries, ranging from 0.4% to 4.2% [4]. Our study considered CSOM as ear discharge lasting more than 2 weeks.

Chronic ear diseases encompass conditions in which there is long standing inflammation in the middle ear [8]. The disease entities include CSOM as well as otitis media with effusion, a condition where there is persistent fluid in the middle ear cavity without signs of suppuration [4, 9].

Consequences of ongoing middle ear infections include hearing impairment (HI), which may result in delays in speech, language, and cognitive skills development, especially if commencing prelingually, [1, 10, 11] and leading to decreased employability in adulthood. In one study in school-going children in Kenya, 64% of schoolchildren with CSOM had HI while only 3.4% of children without CSOM had HI [12]. The only Rwandan study, on prevalence of HI and ear disorders carried out among school children in Kigali, showed that otitis media with effusion which account for the majority of HI in children in the high income countries [13] accounted for only 6.7% of causes of HI among these children. Prevalence of HI was 13.3% [14].

The WHO recommends simple and cost-effective Primary Ear and Hearing Care (PEHC) interventions especially in low and middle income countries (LMICs) to prevent and treat middle ear infections, thus avoiding complications [15]. The concept of PEHC is built on the premise that 50% of causes of hearing impairment are preventable; one of the most preventable of these is middle ear infections. PEHC aims at providing preventive, curative, and supportive measures geared at treating middle ear infections and other causes of hearing impairment at the community level [15]. Implementation of PEHC can be achieved by hitch hiking on existing primary healthcare mechanisms in the community [16].

Considering the recurrent nature of middle ear infections, which may be painless, and the fact that children are most affected yet least able to express themselves, this disease entity tends to be underdiagnosed. Delay to diagnosis may result in a higher rate of postinfection complications. Therefore, it is recommended to avoid risk factors as a way to prevent the first episode [17, 18]. Risk factors include poor hygiene, malnutrition, overcrowding, frequent upper respiratory tract infections, failure to breastfeed, [19, 20], prolonged use of a pacifier, frequenting a day-care centre, and exposure to passive smoking [13, 17].

While clinical records in Rwanda demonstrate a high prevalence of middle ear infections, to the best of our knowledge, Rwanda has no data on prevalence of middle ear infections in children. The aim of this study was to estimate prevalence of middle ear infections among children under five years in Gasabo district in Kigali, Rwanda.

## 2. Methods

### 2.1. Study Design, Setting, and Study Population

This was a cross-sectional study which was conducted in Gasabo district of Kigali city in Rwanda. This district has 486 villages, 229 of which are rural while 257 are urban, with a total population of 530,907 inhabitants (national census, 2012) [21]. There are typically 100–150 households in each village. Villages are supervised by 2 Community Health Workers (cell coordinators) assisted by an assistant cell coordinator. Gasabo district has 17 health centres, 20 health posts, 3 district hospitals, and 1 private referral hospital. It is only in this referral hospital that ear, nose, and throat (ENT) medical services are offered in Gasabo district. The average walking time by foot to a health facility in Gasabo district is 43.6 minutes [22]. The study was a community based cross-sectional study. Study participants were children aged U5 and their parents or guardians.

### 2.2. Sample Size and Data Collection

Using the formula for population studies below, considering a prevalence of 41% among children under 5 in sub-Saharan Africa estimated by Monasta and colleagues [6], we estimated a sample size of 810 parents or guardians and 810 children to obtain a confidence interval of 95%.

(1) $$\begin{array}{c} n1=\frac{z2\times p\left(1-p\right)}{d2}. \end{array}$$

We collected data in March 2016. We used random tables to sample 30 villages from the district. Cell coordinators created a list of households with children U5 from each village. Using random tables, we sampled 27 households from the list of households with children U5 and an additional 3 households for replacement in case we needed to replace a household among the 27. In households with more than 1 child U5, we used Kish tables to sample a child to be examined. Ear examination was performed by a trained research assistant on the sampled child using a Riester Otoscope and findings were recorded in a data sheet modified from the WHO ear and hearing disorders survey tool [23] as shown in Supplementary Material available online at https://doi.org/10.1155/2017/4280583. Ear examination was performed by 2 medical students who had just completed their clerkship in ear, nose, and throat department and also had a refresher training in performing otoscopy and identifying middle ear infections and ear pathologies in recorded images. With the child supported by the parent/guardian, otoscopy was performed by introducing the otoscope into the external auditory canal with one hand while the free hand gently pulled the pinna backwards and downwards. Findings were recorded in the data sheet. We defined middle ear infections as an inflamed eardrum or presence of pus in the external auditory canal or a wet or dry tympanic membrane perforation.

Thereafter, a semistructured questionnaire was administered to the parent or guardian of the sampled child seeking information on demographics of the child, history of middle ear infections, where they sought treatment, and the nature of treatment received, as well as exposure to risk factors.

### 2.3. Quality Control and Ethics

Prior to beginning data collection, we recruited and trained 2 medical students on the study, study tools, and data collection procedure. The students were familiar with ear examination and had a refresher session using recorded images from patients seen locally. A pretest session involving sampling of households, ear examination, and questionnaire completion was conducted in a different district of Rwanda. The questionnaires were then revisited to incorporate observations from the pretest. Parents or guardians of sampled children provided a written consent to be included in the study. The principal investigator conducted otoscopy on 5% of randomly selected children to ascertain accuracy of findings.

Before conducting our study, we sought and obtained approval from both the College of Medicine and Health Sciences Research Ethics Committee of the University of Rwanda and the Makerere University School of Public Health Higher Degrees, Research, and Ethics Committee (HDREC). We obtained a written and signed consent from parents or guardians of sampled children in Kinyarwanda or English to participate in the study. Parents of children found to have an ear infection were encouraged to bring them to seek treatment at a nearest health facility.

### 2.4. Data Analysis

We entered and cleaned data using CSpro 6.2 software and used STATA 13.0 for analysis. Characteristics of the children and examination findings were derived as proportions. Prevalence was obtained by computing proportion of children with middle ear infections among the sampled children. Characteristics of children included age, gender, residence, socioeconomic status (SES), breastfeeding, secondhand smoking, exposure to household cooking smoke, and upper respiratory tract infections. We derived SES using principal component analysis as described by Vyas and Kumaranayake (2006) [24]. We considered and weighted household size and occupancy, types of flooring materials, sanitation facilities, source of water supply, and assets of individual household. This was computed using STATA. The average SES was classified in to low, middle, and high. Chi square tests and logistic regression analysis were used to study associations between independent predictors of ear infection which included socioeconomic status (SES), residence, breastfeeding, secondhand smoking, exposure to household cooking smoke, and upper respiratory tract infections. Bivariate descriptive analysis was conducted to study risk factors after which we progressively built a model to consider subsets of individual risk factors and their interactions with regard to presence of middle ear infections. We applied backward selection to eliminate nonsignificant risk factors taking a significance level of 20% at each level until we only had risk factors whose level of significance was *p* < 0.05.

## 3. Results

The mean age of the 810 children was 28.7 months (SD = 14.1, range 6–59). Females accounted for 50.2% (407) (Table 1). The age group 12–24 months had the highest number of children at 30.4% (237). Almost all children (98.6%) had been breastfed. The mean duration for breastfeeding was 19.8 months (SD = 8.8, range 0–51). Three hundred and thirty-two (41%) children reported to have frequent upper respiratory tract infections and 4.7% (38) were reported to be frequently exposed to passive smoke while 34.4% (279) were frequently exposed to household smoke.

Ear examination revealed that 47 (5.8%) children had an ear infection at the time of the survey. Of the 47 children, 36 (77%) had unilateral disease. Sixteen children had active ear discharge. Of these, 11 (68.7%) had discharge for more than 2 weeks, implying CSOM. Among the children with active ear discharge, 5 parents sought treatment from a medical practitioner and 5 from a traditional healer while another 5 parents did not seek treatment for their children even though they reportedly knew they had an ear infection. One parent bought medicine over the counter. Other ear pathologies found among the 810 children included wax in 13 (1.6%), malformations in 17 (2.1%), and trauma in 4 (0.5%). We did not find any discrepancy in examination findings among the 5% who underwent repeat examination by the principal investigator for quality check.  Table 2 gives a summary of these findings.

When comparing parents' knowledge of whether their child had an ear infection, of 38 parents who said their child had an infection, 29 (76%) were found to be normal on examination while, of 628 parents who reported that their child had no infection, we found 28 (4.5%) of the children had an ear infection. A hundred and forty-four parents did not know if their child had an infection, and yet, of these, 10 children were found to have an ear infection. It is noteworthy that 32 (4%) children had a tympanic membrane perforation or an active discharge yet only 16 (2%) children had been reported to have ear infection by their parents.

Breastfeeding, regardless of duration, did not show any association with developing middle ear infections, and neither did gender, SES, exposure to secondhand smoke, or respiratory tract infections.

On bivariate analysis, a child was less likely to develop middle ear infections if they lived in an urban setting (OR = 0.52, 95% CI: 0.29–0.96) but more likely if exposed to household smoke (OR = 2.54, 95% CI: 1.18–5.46). Parents who had attained secondary education and above were less likely to have children suffering from middle ear infections. These findings were significant at both bivariate (OR 0.17, 95% CI: 0.06–0.54) and multivariate analysis (OR 0.22, 95% CI: 0.67–0.73).

We found a strong discrepancy between awareness of parents about their child having a middle ear infection and our examination findings. Parents were 0.2 times less likely to recognise that their child was having an ear infection when they reported that they knew about middle ear infections (adjusted OR 0.18, 95% CI: 0.07–0.46). On the other hand, they were 0.3 times less likely to recognise that their child was having an ear infection when they reported that they did not know about ear infections (adjusted OR 0.28, 95% CI: 0.09–0.84). These findings were significant on both bivariate and multivariate levels. More details can be found in Table 3.

## 4. Discussion

This is one of the first studies to investigate the prevalence of middle ear infections in Rwanda. We found a prevalence of middle ear infections of 5.8%, of which 4.0% had chronic suppurative otitis media (CSOM). This is comparable to the prevalence of 0.4–4.2% reported by the WHO for Africa although global estimates range between 0.4 and 46%, with the Australian Aborigines having the highest prevalence [4, 7]. The prevalence of CSOM in a recent study in Kenya was 1.5%, similar to Gambia and Tanzania [25], but rises to 7% among school-going children in a study in Nigeria [26]. The WHO classifies national prevalence of CSOM as low (2%), high (2–4%), and highest if >4% [7]. Thus based on this, the prevalence of middle ear infections in this district of Kigali in Rwanda is considered high. Differences in the definition of CSOM account for the wide variation of reported prevalence among countries even in the same region [4, 25, 27, 28]. Moreover, this variation could depend on presence of different risk factors in different regions [27, 28].

Our study found no significant gender differences in prevalence of middle ear infections contrary to reports of female preponderance in other studies [26, 29, 30]. Ear infection was commonest among infants compared to the older kids. Thirty of the children with middle ear infections (64%) were aged 3 years and below. Middle ear infections are known to mostly affect young children. These results are similar to findings from other studies reporting that most children will have had an ear infection by their third birthday [9, 13]. This is attributed to immature immunity coupled with anatomical characteristics of the Eustachian tube in young children as opposed to adults [13]. Educating parents and caregivers on middle ear infections and raising their index of suspicion especially in the vulnerable age group of children aged below 3 years may help to pick and treat middle ear infections early to avoid complications.

Our study did not find a relationship between SES and middle ear infections but found that children living in rural areas were more likely to have middle ear infections than those in urban environments. Many studies show that poor SES is a risk factor for developing middle ear infections, especially considering overcrowding, malnutrition, and poor hygiene [31–33]. However, while results showed a tendency of decreased middle ear infections with improvement in SES, this was not statistically significant. This could be explained by overall low awareness of middle ear infections among parents regardless of their level of education as well as poor health-seeking practices implying that all children have low exposure to treatment as was shown by our own research.

Education was the only risk factor that was significant in both bivariate and multivariate analysis. Children were less likely to have a middle ear infection if their parent or guardian was more educated compared to being uneducated. Protective effects of parental education have been described in other studies [20, 34]. Thus there is a need to provide health education especially targeting lowly or uneducated parents whereas at the same time strengthening education and raising awareness in schools.

Our study found no relationship of middle ear infections and breastfeeding regardless of duration yet breastfeeding is known to be protective for middle ear infections [9, 20, 32]. Moreover, there was no difference in middle ear infections among the breastfed and nonbreastfed children. A possible explanation for this is that in our study there were only a few children who were never breastfed. Breastfeeding is nearly universal in Rwanda. A similar finding was reported by Zhang and colleagues (2014) in their meta-analysis where none of the studies they included showed a correlation between breastfeeding and middle ear infections [35]. Karunanayake and colleagues (2016) compared incidences of middle ear infections in children in 2 rural communities and found that children were less likely to develop middle ear infections if they were breastfed for at least the first 3 months [20]. Being a multifactorial condition, sheer lack of breastfeeding in absence of other risk factors may not increase risk of developing middle ear infections.

Frequent upper respiratory tract infections, exposure to household smoke and passive smoke, and other factors increase the risk of developing middle ear infections by supporting colonization with pathogens [20, 31]. There was no difference in middle ear infections in children who always had upper respiratory tract infections compared to those who never had these infections. While this is a surprising finding given the anatomical relations and spread of infection in children [13, 20], it is difficult to explain why these infections were not a risk factor among our population. We however postulate that parents could have exaggerated the presence of respiratory tract infections or confused them with simple allergies in children.

We found that children exposed to secondhand household smoke were 2.5 times likely to develop middle ear infections. The effect of smoke on mucociliary function and consequent risk for middle ear infections has been documented [9, 20, 35]. In agreement with our study, Lasisi and colleagues (2007) found that indoor-cooking was a risk factor for CSOM [36]. The government should scale up the existing strategies to reduce exposure to household smoke such as utilization of cooking stoves to benefit even those with low SES and those living in rural areas [37, 38].

Pain was the most common symptom reported by parents who reported that their children had middle ear infections. Symptoms of middle ear infections vary depending on stage of the disease. Acute otitis media presents with pain and fever and hearing loss at times while CSOM presents with ear discharge and hearing loss [4, 9]. Hearing loss was not a common complaint. This is contrary to other studies where hearing loss is the main complaint for CSOM [7, 26, 29, 32, 39]. Our previous studies found that young children are unlikely to report hearing loss. Moreover, due to low awareness, parents are unlikely to detect hearing loss until children attain school age. Usually, affected children present with delays in speech and language development and poor performance in school [2, 7, 40, 41]. Moreover, we found that only 1 in 4 parents correctly suspected that their child had an ear infection while the majority of the parents suspected an ear infection when it did not exist. To address this, early childhood hearing screening should be implemented so as to pick and treat children with infections and those with hearing loss.

The majority of our subjects with ear disease had CSOM. Interestingly, only 31% sought treatment at a health facility. This represents risky health-seeking practices. Poorly managed or untreated acute middle ear infections result in chronically discharging ears and increase the risk of hearing loss [20, 41, 42]. Moreover, alternative therapies for treatment for middle ear infections have been reported in Rwanda and in other parts of the world [29, 43, 44]. Effectiveness of these therapies is an issue of debate. However, reliance on alternative therapies for treatment of middle ear infections puts the patient at risk of disease progression and development of complications as shown in our other studies. This practice should be shunned. Factors associated with risky health-seeking behaviour include lack of awareness, poverty, beliefs, and unfavorable health systems [19, 29, 32, 40, 44].

Results from this study put forth evidence that middle ear infections in children should be a public health concern. Parents are unlikely to know that their children have middle ear infections and this results in delays in seeking care. Moreover, risk factors for middle ear infections can all be mitigated through raising awareness, educating families and healthcare workers on health promotion strategies, and early diagnosis and treatment. Therefore, it is imperative that the government takes a lead in curbing the prevalence of middle ear infections in children since this is a significant barrier towards achieving education for all.

This study had a number of methodological strengths but also weaknesses. The strength of the study is being one of the first to document prevalence of middle ear infections in Rwanda in any setting, thus setting a baseline for future studies. In addition, we also had a large number of mothers whom we interviewed and kids whom we assessed. However, the study also has limitations. The proportion of children with ear infection was small, impeding us from studying other possible correlations. The operational definition included hyperemic tympanic membrane and presence of a perforation, both of which could not necessarily be caused by an infection; infections are the most common cause. Moreover, this was a cross-sectional study and responses were estimates made by parents and could not be quantified and there could have been a recall bias for some of the responses. Further research to establish risk factors for middle ear infections in Rwandan children is recommended.

## 5. Conclusion

Middle ear infections remain a public health problem in Rwanda. The prevalence is far higher than the threshold used by the World Health Organization to qualify as a public health problem. However many parents were not aware of its presence in the affected children. Low education levels and being exposed to household smoke were risk factors whereas living in an urban area seemed to be protective. We also found that whenever children fell ill, many parents preferred use of traditional medicine. There is a need to raise awareness of parents about ear infection and to promote early care seeking from qualified health workers.

## Supplementary Material

Modified version of the WHO ear and hearing disorders survey tool we used to collect data.

## Figures and Tables

**Table 1:** Characteristics of children.

Characteristics	Frequency	%
Gender		
Males	403	49.8
Females	407	50.2
Age (months)		
≤12	115	14.2
13–24	246	30.4
25–36	223	27.5
37–48	147	18.2
≥49	79	9.8
SES		
Low	383	47.3
Middle	332	41.0
High	95	11.7
Level of education		
No education	50	6.2
Primary	465	57.5
Secondary	220	27.2
Vocational	74	9.2
Upper respiratory tract infections		
Never	21	2.6
Sometimes	457	56.4
Always	332	41.0
Breastfeeding		
Never	11	1.4
<12 months	143	17.7
12–24 months	316	39.0
25–36 months	328	40.5
>36 months	12	1.5
Household smoke		
Never	307	37.9
Sometimes	224	27.7
Always	279	34.4
Passive smoking		
Never	669	82.6
Sometimes	103	12.7
Always	38	4.7
Residence		
Rural	378	46.7
Urban	432	53.3
Accuracy of parents' knowledge		
Knowing about infection	561	69.3
Not knowing about infection	249	30.7

**Table 2:** Characteristics of children with active discharge.

Characteristics	*N* = 16
*Duration of discharge*	
2 weeks	5 (31.3%)
>2 weeks	11 (68.8%)
*Course of discharge*	
First time	4 (25%)
On and off	11 (68.8%)
Constant	1 (6.3%)
*Treatment options*	
Traditional medicine	5 (31.3%)
Prescription medicine	5 (31.3%)
Self-medicated	1 (6.3%)
Not treated	5 (31.3%)

**Table 3:** Independent predictors of ear infection.

	Number of children without middle ear infections	Children with middle ear infections (*n* = 47)	Crude OR (CI)	Adjusted OR (CI)
Gender				
Males	379 (49.7%)	24 (59.1%)	1	1
Females	384 (50.3%)	23 (48.9%)	0.95 (0.52–1.71)	0.94 (0.52–1.73)
Age (months)				
≤12	107 (14.2%)	8 (17%)	1	1
13 –24	232 (30.4%)	14 (29.8%)	0.81 (0.33–1.98)	0.79 (0.36–1.97)
25–36	215 (28.1%)	8 (17%)	0.50 (0.18–1.36)	0.44 (0.16–1.23)
37–48	139 (18.2%)	8 (17%)	0.77 (0.28–2.12)	0.66 (0.23–1.87)
≥49	70 (9.2%)	9 (19.2%)	1.72 (0.63–4.67)	1.41 (0.51–3.98)
SES				
Low	359 (47.1%)	24 (51.1%)	1	1
Middle	312 (40.9%)	20 (42.5%)	0.96 (0.52–1.77)	1.31 (0.67–2.57)
High	92 (12.1%)	3 (6.4%)	0.49 (0.14–1.66)	0.94 (0.25–3.58)
Level of education				
No education	43 (5.6%)	7 (14.9%)	1	1
Primary	434 (57%)	31 (66%)	0.44 (0.18–1.01)	0.45 (0.18–1.11)
Secondary	214 (28.1%)	6 (12.8%)	0.17 (0.06–0.54)^**∗**^	0.22 (0.67–0.73)^**∗∗**^
Vocational	71 (9.3%)	3 (6.4%)	0.26 (0.06–1.06)	0.41 (0.89–1.85)
Breastfeeding (months)				
Never	10 (90.9%)	1 (9.1%)	1	1
<12	133 (93.0%)	10 (7.0%)	0.75 (0.09–6.48)	0.51 (0.5–5.41)
12–24	293 (92.7%)	23 (7.3%)	0.78 (0.10–6.40)	0.52 (0.06–4.76)
25–36	316 (96.3%)	12 (3.7%)	0.38 (0.05–16.54)	0.23 (0.02–2.26)
>36	11 (91.7%)	1 (8.3%)	0.91 (0.05–16.5)	0.39 (0.02–8.60)
Household smoke				
Never	297 (38.9%)	10 (21.3%)	1	
Sometimes	209 (27.4 %)	15 (31.9%)	2.13 (0.94–4.84)	1.78 (0.73–4.36)
Always	257 (33.7%)	22 (46.8%)	2.54 (1.18–5.47)^**∗**^	1.83 (0.65–3.93)
Passive smoking				
Never	634 (83.1%)	35 (74.5%)	1	1
Sometimes	95 (12.5%)	8 (17%)	1.53 (0.69–3.39)	1.26 (0.55–2.92)
Always	34 (4.5%)	4 (8.5%)	2.13 (0.72–6.34)	1.83 (0.58–5.65)
Residence				
Rural	349 (45.7%)	29 (61.7%)	1	1
Urban	414 (54.3%)	18 (38.3%)	0.52 (0.29–0.96)^**∗**^	0.17 (0.33–0.46)
Accuracy of parents' knowledge compared to examination findings				
Not knowing about infection	8 (1.1%)	8 (17%)	1	1
Having infection	750 (98.3%)	38 (80.9%)	0.15 (0.07–0.35)^**∗**^	0.18 (0.07–0.46)
Not having infection	5 (0.7%)	1 (2.1%)	0.24 (0.09–0.64)	0.28 (0.09–0.84)

^**∗**^Significant at bivariate analysis; ^**∗∗**^significant at multivariate analysis.
