# Supplementary material for: Prevalence of Middle Ear Infections and Associated Risk Factors in Children under 5 Years in Gasabo District of Kigali City, Rwanda
Source: Int J Pediatr. 2017 Dec 3;2017:4280583. doi: 10.1155/2017/4280583 (PMC5733628; doi:10.1155/2017/4280583)
Supplement: Supplementary file 1 — Modified version of the WHO ear and hearing disorders survey tool we used to collect data. [file 4280583.f1.pdf]

Prevalence of ear infections among children under five years

Identification

Sector name................................/....../...../ Cell name ..../....../....../

Village name ..../....../...../ Household number ….....................…/....../....../

Number of children aged >6 – 59 months………………..

Data collection tool

1. **Identification of the subject**

Age (months)…………

Gender: 1. Male ☐ 2. Female ☐

Lives with: Father ☐ Mother ☐ Both ☐

Guardian ☐ → **Q 2.3**

2. **Socio-economic data**

2.1 Mother's education:

1. None ☐
2. Primary ☐
3. Secondary ☐
4. Tertiary ☐
5. Vocational ☐

Occupation
1. **Farmer**
2. **Salaried job**
3. **Casual laborer**
4. **Self employed**
5. **Other job**
6. **Unemployed**

2.1.1 Occupation……………………………………………

2.2 Father's education

1. None ☐
2. Primary or less ☐
3. Secondary ☐
4. Tertiary ☐
5. Vocational ☐

2.2.1 Occupation……………………………………………….

2.3 Education level of guardian

1. None ☐
2. Primary or less ☐
3. Secondary ☐

4. Tertiary ☐
5. Vocational ☐

3. **Household size**
   1. How many rooms does this house have?.......................
   2. How many people live in this house?............................

4. **Household conditions - toilet**
   1. Flush toilet
   2. Traditional latrine floor
   3. Traditional latrine without a floor
   4. Shared
   5. No toilet

5. **Source of drinking Water**
   1. Piped inside dwelling
   2. Communal standpipe
   3. Tapped Spring water
   4. Untapped spring water
   5. Rain water
   6. River, lake, Dam
   7. Somewhere else

6. **Flooring material**
   1. Earth, smoothed mud, Sand
   2. Dung
   3. Cement
   4. Bricks
   5. Stones
   6. Wood
   7. Tiles
   8. Other…………………………………………………………………

7. **Ownership of durable goods/livestock** **Number**
   1. Mobile phone ☐
   2. Radio ☐

3. TV
4. Bicycle
5. Motorbike
6. Car
7. Truck
8. Refrigerator
9. Computer
10. Cow
11. Goats
12. Sheep
13. Pigs
14. Rabbits
15. Birds

8. **History of child's illness**

8.1 Does your child have Ear discharge today?

1. Yes
2. No
3. Unknown

If No/ unknown, → Q **8.1.2**

8.1.1 If Yes: Duration of discharge

1. Less than 2 weeks
2. More than two weeks
3. Unknown duration

8.1.1.1 Course of discharge

1. It is the first time
2. It is on and off
3. It is constant

8.1.1.2 What treatment did they give your child when he/she was unwell?

1. Traditional medicine
2. Medicine given in health facility
3. Self medicated from pharmacy

4. Underwent Surgery
5. He/ she was not treated
6. Others……………………………….

8.1.2 If No: Has the child ever had ear discharge?

1. Yes
2. No
3. I don't know

9. Other ear symptoms
    1. Hearing loss
    2. Pain
    3. Skin eruptions
    4. Others………………………………
    5. None
    6. I don't know

10. How often is your child exposed to household smoke?
    1. Never 2. Sometimes 3. Always

11. How often is your child passive smoking
    1. Never 2. Sometimes 3. Always

12. How long did your child breastfeed?
    1. Never 2. …………………..months

13. How frequently does your child suffer from colds/cough?
    1. Never
    2. Sometimes
    3. Always

14. **Examination findings**

	Right ear		Left ear	
10.1 Ear Pain	Y	N	Y	N
10.2 Normal auricle	Y	N	Y	N
10.3 Congenital malformations	Y	N	Y	N
10.4 Trauma recent/Old	Y	N	Y	N

**10.5 External ear canal**	**Right ear**		**Left ear**	
1. Normal	Y	N	Y	N
2. Congenital malformations	Y	N	Y	N
3. Trauma recent/Old	Y	N	Y	N
4. Inflammation	Y	N	Y	N
5. Wax	Y	N	Y	N
6. Foreign body	Y	N	Y	N
7. Otorrhoea	Y	N	Y	N
8. Fungi	Y	N	Y	N

**10.6 Ear drum**	**Right ear**		**Left ear**	
1. Normal	Y	N	Y	N
2. Perforation	Y	N	Y	N
3. Dullness or Retraction	Y	N	Y	N
4. Red	Y	N	Y	N
5. Bulging	Y	N	Y	N
6. Not visible	Y	N	Y	N

**10.7 Middle Ear**	**Right ear**		**Left ear**	
1. Normal TM	Y	N	Y	N
2. Otorrhoea	Y	N	Y	N
3. Dry	Y	N	Y	N

4. Others (state affected ear) ....

Thanks for your time
